# A high-resolution time-depth view of dimethylsulphide cycling in the surface sea

**DOI:** 10.1038/srep32325

**Published:** 2016-08-31

**Authors:** S.-J. Royer, M. Galí, A. S. Mahajan, O. N. Ross, G. L. Pérez, E. S. Saltzman, R. Simó

**Affiliations:** 1 Institut de Ciències del Mar, CSIC, Passeig Marítim de la Barceloneta 37-49, 08003 Barcelona, Catalonia, Spain; 2 Department of Oceanography, School of Ocean and Earth Science and Technology, University of Hawaii at Mānoa, 1950 East-West Road, 96822, Honolulu, Hawaii, USA; 3 Takuvik Joint International Laboratory and Québec-Océan, Université Laval, G1V OA6, Québec, QC, Canada; 4 Indian Institute of Tropical Meteorology (IITM), Pashan Road, 411 008, Pune, India; 5 Aix-Marseille University, CNRS, University of Toulon, IRD, MIO UM 110, 13288, Marseille, France; 6 Instituto INIBIOMA (CRUB Comahue, CONICET), Quintral 1250, 8400 S.C. de Bariloche, Rio Negro, Argentina; 7 University of California, Earth System Science Department, 3200 Croul Hall St, Irvine, California, 92697, United States

## Abstract

Emission of the trace gas dimethylsulphide (DMS) from the ocean influences the chemical and optical properties of the atmosphere, and the olfactory landscape for foraging marine birds, turtles and mammals. DMS concentration has been seen to vary across seasons and latitudes with plankton taxonomy and activity, and following the seascape of ocean’s physics. However, whether and how does it vary at the time scales of meteorology and day-night cycles is largely unknown. Here we used high-resolution measurements over time and depth within coherent water patches in the open sea to show that DMS concentration responded rapidly but resiliently to mesoscale meteorological perturbation. Further, it varied over diel cycles in conjunction with rhythmic photobiological indicators in phytoplankton. Combining data and modelling, we show that sunlight switches and tunes the balance between net biological production and abiotic losses. This is an outstanding example of how biological diel rhythms affect biogeochemical processes.

The knowledge gained about the trace gas dimethylsulphide (DMS) and its main biological precursor, the algal osmolyte dimethylsulphoniopropionate (DMSP), over the last 40 years makes these compounds some of the best-studied organic substances in the world’s oceans^1^,^2^. Oceanic emissions of DMS play a fundamental role in marine aerosol formation and growth^3^,^4^, and in the cycling of sulphur between the oceans, atmosphere, and continents^5^. DMS also plays a role in chemical ecology as an aerial olfactory signal sensed by marine mammals^6^, turtles^7^ and birds (ref. 8 and refs therein), and as an underwater infochemical for marine plankton^9^,^10^,^11^, with important ecological consequences^10^,^12^,^13^. The hypothesized role of DMS in a plankton-clouds-climate feedback loop, has stimulated considerable discussion and remains controversial^3^,^14^,^15^.

Oceanic DMS concentration depends on the interplay of many processes: DMS is produced mainly by enzymatic degradation of the phytoplankton osmolyte DMSP with involvement of the entire planktonic food web^1^, and is lost by ventilation into air, bacterial catabolism, and photochemical oxidation^16^,^17^. A number of studies have addressed the seasonality of DMS concentration in relation to solar radiation, vertical mixing, nutrient availability and associated biological succession (e.g., refs 17, 18, 19, 20, 21, 22, 23). A general pattern emerges by which DMS concentration is higher in summer and lower in winter regardless of latitude and productivity^24^,^25^. This has prompted to computationally map global DMS distribution as a sole function of solar radiation and underwater optics on the seasonal scale^15^,^24^,^25^, thus providing the strongest support hitherto to the original hypothesis^3^ that the ocean responds to solar radiation by proportionally emitting aerosol-forming, cloud-seeding DMS^24^. Whether DMS responds to solar radiation also at meteorological scales (i.e., hourly to weekly) and, if so, through what mechanisms, remains unknown.

Few studies have addressed the short-term variability of DMS on time scales of hours to weeks across phases of natural and fertilized phytoplankton blooms^26^,^27^,^28^,^29^,^30^. Even more scarce are studies addressing the causes of short-term DMS variability anywhere in the oligotrophic oceans^31^,^32^,^33^. Investigating issues such as DMS resilience to perturbation, or its coupling to plankton photo-physiology, require observations of vertical DMS distribution and dynamics. One of the impediments to such studies is the difficulty in making trace gas measurements at frequencies sufficient to resolve variability due to changes in meteorological, hydrological and biogeochemical conditions. Vertical profilers have been used to make high frequency measurements of seawater temperature, density or chlorophyll *a* (chl*a*) fluorescence but traditional analytical methods for DMS take several minutes for a single measurement. This study takes advantage of high frequency mass spectrometric and optical measurements, coupled to continuous vertical sampling with a recently developed profiler to allow a view of upper ocean DMS cycling at unprecedented resolution. Here we report results from two Lagrangian studies conducted in open oligotrophic waters of the western Mediterranean Sea illustrating the complex response of DMS cycling to changes in meteorology, solar radiation, and phytoplankton photophysiology.

## Results

### Sea surface data and meteorological forcing

**Figure 1:**
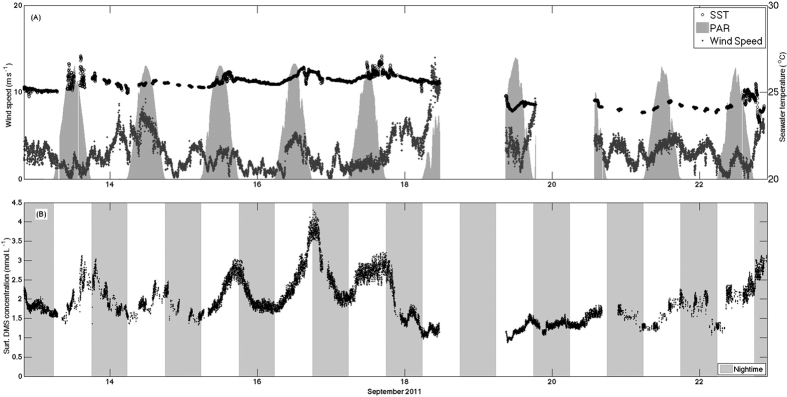
Lagrangian series of DMS and physical forcing in the September 2011 cruise. (**A**) Times series of wind speed (m s^−1^ – dark grey); sea surface temperature (°C – black circles); and solar radiation in light grey (scaled; maximum irradiance of 1770 μmol photons m^−2^ s^−1^). (**B**) Time series of DMS surface concentrations (nmol L^−1^). The shaded bars represent nighttime. The large data gaps on days 18–20 were due to a storm and instrumental problems.

In September, continuous data recording over 10 days of tracking a water mass showed a remarkable picture of the coupling between atmospheric physical forcing and biogeochemistry of the sea surface (Fig. 1). Wind speeds were generally low (average winds < 5 m s^−1^), with diurnal variations due to basin-scale sea breeze circulation. There were two episodes of increased winds. On 14 September winds increased to 7–8 m s^−1^ for almost 12 hours and on 18–19 September elevated winds associated with a storm exceeded 14 m s^−1^ and obliged to stop the cruise for 30 hours. Calm winds and clear skies followed the storm for the last 2.5 days of the cruise (Fig. 1A). Sea surface temperature (SST) exhibited a diurnal cycle of 1–2 degrees, in response to high daytime solar irradiance and nocturnal cooling. Mean SST levels gradually increased over the first 5 calm and sunny days, followed by cooling by nearly 2 °C during the storm due to heat loss and vertical mixing.

Surface DMS concentration time series also exhibited a strong diurnal cycle. DMS levels increased during the day and decreased at night, with a minimum near dawn and maximum around dusk (Fig. 1B). Mean surface DMS concentrations (1.5–4 nmol L^−1^) increased gradually on calm sunny days, and decreased by about 70% during the stormy period on 18–19 September (Fig. 1B). On the subsequent sunny days, DMS recovered gradually to pre-storm levels. Before the windstorm, the dusk – dawn difference reached 2 nmol L^−1^, roughly a doubling in concentration over the course of the day. The diurnal cycle was disrupted by the storm, and subsequently recovered as DMS levels increased on the next few days. There is a striking similarity in the evolution of sea surface temperature (SST) and DMS in response to meteorological perturbation, vertical mixing, and re-stratification.

**Figure 2:**
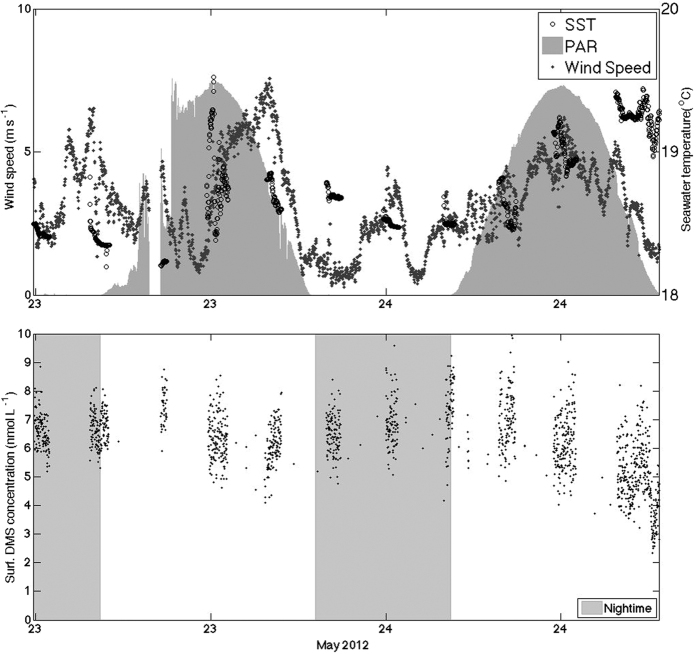
As in Fig. 1 but for the May 2012 cruise. Temperature and DMS data are not continuous because they were collected with the vertical profiler, which had to be stopped and drawn out of the water every time a CTD cast was taken.

During a brief, 2-day cruise in late May, wind speed was lower than during the September cruise, with a minimum of 0.27 m s^−1^ and a maximum of 7.6 m s^−1^ (Fig. 2A). DMS concentrations (5–8 nmol L^−1^) were considerably higher than in September, in agreement with the typical seasonality in the region^20^. The two sunny days were characterized by a strong day-night pattern in surface DMS concentration (Fig. 2B, inset). However, the diurnal cycle in May was opposite in phase to that in September, with nighttime increase and daytime decrease (i.e., maximum concentration around dawn, minimum at dusk).

### Phytoplankton composition and DMSP

**Figure 3:**
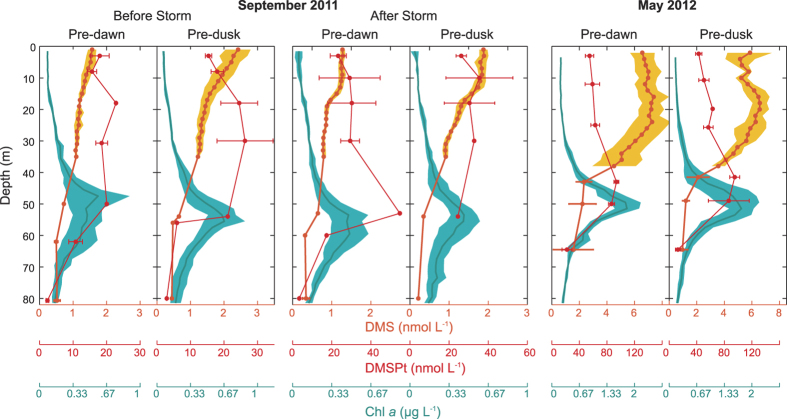
Vertical patterns. Averaged vertical profiles from the surface to below the deep chlorophyll maximum (DCM) for DMS (nmol L^−1^), DMSP_t_ (nmol L^−1^) and Chl-*a* (μg L^−1^; fluorescence profiles calibrated with extracted chl*a* measured in discrete bottles). DMS below 35 m and DMSP_t_ data are from discrete samples from CTD rosette. DMS above 35 m and chl*a* profiles are 30 seconds means of measurements from the continuous vertical profiler, and standard deviations are indicated as shaded areas. Pre-dawn and pre-dusk profiles are shown for (**A**) 13–14 September before storm, (**B**) 21–22 September after storm, (**C**) May. The depth of the mixing layer is indicated in Fig. 4.

According to cell counts and taxonomic pigments, surface phytoplankton in September were dominated by dinoflagellates, *Synechococcus* and prymnesiophytes. Phytoplankton at the deep chl*a* maximum (55 m approx.) were dominated by *Prochlorococcus*, dinoflagellates and prymnesiophytes. In May, the surface assemblage was rather similar to that in September, but the deep chl*a* maximum assemblage was dominated by prymnesiophytes, dinoflagellates, *Synechococcus* and pelagophytes. Surface chl*a* concentrations were similar during the two cruises (nearly 0.1 μg L^−1^), but the concentration at the deep chl*a* maximum was 1.5–2 times as high in May (Fig. 3). Total DMSP (particulate + dissolved; DMSPt) concentrations in September and May ranged 20–40 nM and 50–100 nM, respectively.

### Vertical profiles of DMS, DMSP and chl*a*

Vertical profiles reveal a more complete picture of the processes controlling surface DMS variability. Figure 3 shows changes in vertical profiles associated with the diurnal cycles of DMS, DMSP and chl*a*. In September, DMS was higher at the surface, and the increase at dusk extended from the surface to 15–20 m, i.e., beyond the bottom of the mixing layer into the thermocline. DMSPt concentrations also increased between dawn and dusk, particularly so at the thermocline (20–50 m), within the water layer that in oligotrophic waters typically exhibits the maximum primary production rates. Chl*a* concentrations showed a clear deep maximum around 50–60 m, and increased over daytime through most of the water column. In May, a slight chl*a* concentration increase from dawn to dusk was only noticeable at the deep maximum (50 m). DMSPt behaved similarly at depth but the concentration slightly decreased at surface during daytime. Mixed-layer DMS also decreased during daytime.

The profiles described above portray the typical open ocean vertical distributions in oligotrophic waters^18^, where chl*a* peaks at depth (at the bottom of the seasonal thermocline), DMSPt peaks from right above the chl*a* peak to right below the mixed layer, and DMS peaks up in the mixed layer, or right below.

### High-resolution time-depth profiles

**Figure 4:**
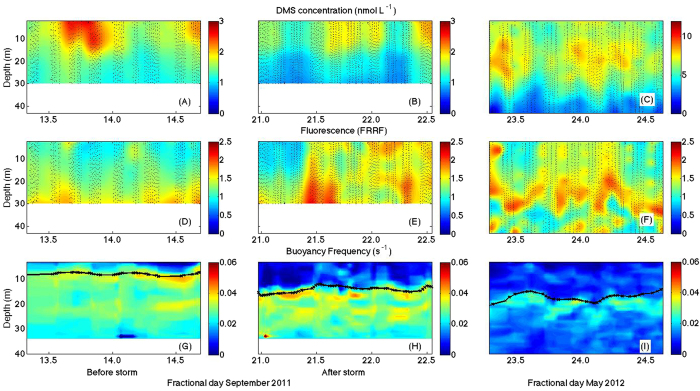
High-resolution time-depth distributions during the intensive Lagrangian studies. The positions of the original data are shown as black dots in the foreground. Colors show data interpolation over 1 m and 1 min. (**A–C**) DMS concentrations (nmol L^−1^), (**D–F**) chlorophyll *a* fluorescence from the FRRf, (**G–I**) buoyancy frequency (s^−1^). Black lines on the buoyancy plots represent the mixing layer depth calculated from a 0.05 kg m^−3^ departure from the density at a reference depth of 2 m.

The use of continuous data recording in continuous profiling mode permitted monitoring the vertical changes of target variables at much higher temporal resolution than CTD-rosette sampling profiles. DMS and *in vivo* fluorescence measured with an FRRf (as a proxy for chl*a*) were recorded (Fig. 4). Unfortunately there was no method yet for continuous measurements of DMSPt. In the calm days of September before the storm, surface waters were mixed to only 5–10 m, the depth at which the pycnocline and thermocline began. Wind stress and heat loss during the storm increased surface water turbulence and instability, deepening the mixing layer. Before the storm, the deep chl*a* maximum (Fig. 3) could barely be detected in the continuous fluorescence profiles (Fig. 4). After the storm there was an increase of phytoplankton fluorescence at shallower depths, which was not captured in the bottle profiles because of the coarse depth resolution. In both cases, high-resolution fluorescence confirmed the discrete chl*a* profiles and was higher at depth during the day, with a weaker diel pattern at surface. The DMS distribution was inverted relative to that of fluorescence. DMS accumulated in surface waters (<20 m) and increased in the afternoon towards dusk. Days after the windstorm, DMS had recovered its diurnal cycle within the upper 30 m.

In May, vertical turbulent mixing was deeper (15–20 m, as shown by the mixing layer depth line in Fig. 4). High-resolution fluorescence revealed the occurrence of a well-defined maximum at around 30 m, which was not sampled in the bottle profiles. This chl*a*-rich layer exhibited oscillations, likely due to internal waves, but no clear diel pattern. By contrast, at the surface fluorescence was higher during the night and lower during the day, a phenomenon that might be indicative of phytoplankton photoacclimation cycles^34^. DMS peaked at the very surface and surprisingly, just below the mixed layer. The diel pattern of nighttime increase and daytime decrease that was observed at surface (Fig. 2B) had its maximal amplitude at around 20 m depth and propagated to a depth of at least 30 m.

### Photobiological clocks

**Figure 5:**
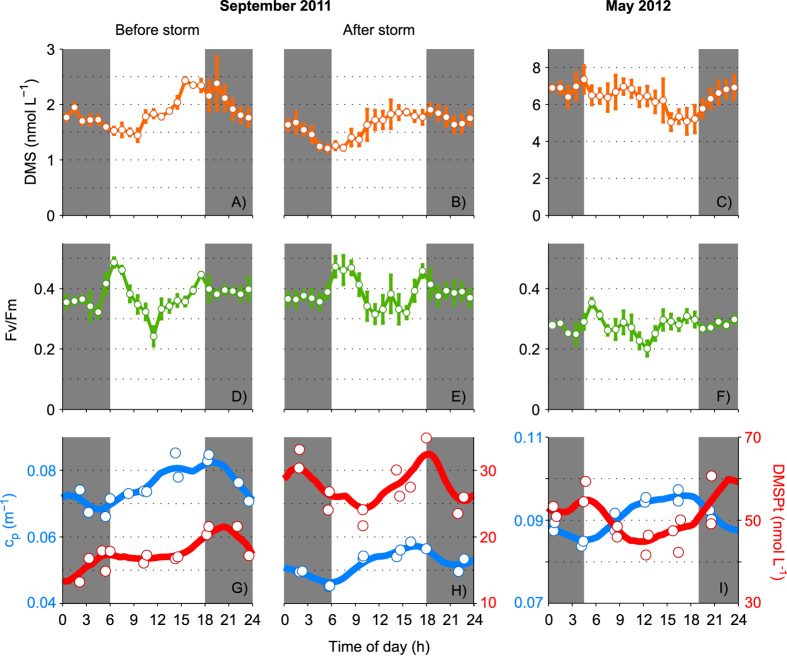
Characteristic diel cycles of DMS(P) and photobiological indicators in surface waters. High-resolution DMS concentration (**A–C**) and photosystem II efficiency (Φ_PSII_, **D–F**) binned to hourly frequency, plus total DMSP (DMSPt) concentration and beam attenuation due to particles (c_p_) measured every four hours from CTD-Niskin bottle casts (panels G–I). The data represent upper mixed-layer averages from which the underlying two-day trend (if any) has been removed. Lines in **G–I** are spline fits smoothed with 4-h running means.

The fact that the diel variation series of surface DMS concentrations had dawn and dusk as the minima/maxima suggests the involvement of photobiological processes with diel rhythms. Figure 5 compares diel cycles of hourly-averaged DMS and an indicator of phytoplankton’s photosystem II (PSII) efficiency (Φ_PSII_). The latter displayed two features typically observed in stratified surface waters: 1) a midday depression, caused by high irradiance and the subsequent increase in non-photochemical quenching and possible photosystem damage, and 2) pre-dawn and pre-dusk peaks, related to changes in the photosystem electron acceptor pools. DMSPt and the beam attenuation coefficient due to particles at 660 nm (c_p_), also displayed diel periodicity. A regular c_p_ cycle with dawn minima and pre-dusk maxima was found in all three intensive sampling periods. This feature is ubiquitous in the open ocean and stems mainly from the increase in cell size of phytoplankton due to photosynthesis^35^,^36^. Since c_p_ is most responsive to changes in the particle population of sizes ranging 0.5–20 μm, and especially around 5 μm^35^, it is representative of the carbon biomass of nanophytoplankton responsible for DMS(P) production. The nighttime c_p_ decrease was probably related to synchronized cell division at night^37^ and grazing losses. The DMSPt cycle resembled that of c_p_ but was lagged in time and less repeatable: after an early morning plateau or decrease, DMSPt increased steadily until dusk or into the evening, and decreased at night. Thus, the trend reversal points of the diel DMS pattern (i.e., the times at which net production changed into net consumption or vice versa) coincided with switching points marking the diel phasing of phytoplankton physiology and cell growth, and to a lesser extent to changes in the sign of net DMSPt variations.

### Computed turbulent diffusivity and abiotic DMS losses

The General Ocean Turbulence Model (GOTM) was used to compute vertical fields of the turbulent eddy diffusivity over time for the three intensive studies (Fig. S2). The model was forced with meteorological data recorded during the cruise.

**Figure 6:**
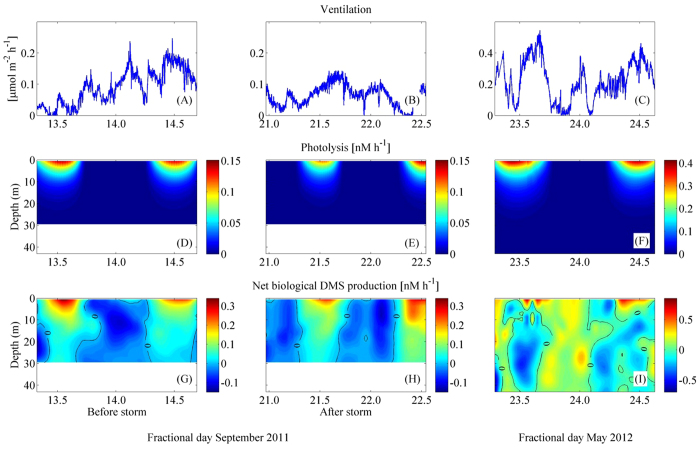
Computed rates for DMS cycling processes in the three intensive studies. (Top) Ventilation rate (μmol m^−2^ h^−1^). (Middle) Photolysis rate (nmol L^−1^ h^−1^). (Bottom) Net biological production (nmol L^−1^ h^−1^).

Turbulent transport of DMS into and out of the mixing layer (i.e. through the top of the pycnocline) occurred mainly during short episodes (2–4 h long) associated to nighttime convection and/or wind outbursts. Downward DMS transport events occurred on 14 and 21 September, early morning (ca. −1 μmol m^−2^ h^−1^) and on 23 May early morning (−2.3 μmol m^−2^ h^−1^). Upward DMS transport events were observed on 22 September midday (2.6 μmol m^−2^ h^−1^) and 24 May midnight (2.5 μmol m^−2^ h^−1^). The average transport fluxes across the base of the mixing layer (defined with the Δσ_t_ = 0.05 kg m^−3^ criterion) were smaller than ±0.07 μmol m^−2^ h^−1^in the three intensive studies, which represented contributions to the volumetric DMS budget in the mixing layer smaller than ±0.008 nM h^−1^. Calculated DMS ventilation fluxes ranged up to 0.27 μmol m^−2^ h^−1^ in September and up to 0.5 μmol m^−2^ h^−1^ in May (Fig. 6). Profiles of DMS photolysis rates were estimated from incubations under controlled conditions and computationally transported *in situ* by use of underwater UV radiation fields. In September, photolysis rates at noon under the surface reached 0.13 nmol L^−1^ h^−1^, and decreased with depth to negligible values below 20 m. In May, subsurface photolysis rates at noon reached 0.40 nmol L^−1^ h^−1^, and became negligible at around 30 m deep (Fig. 6).

### Diel cycles of net biological DMS production (NPBIO)

The turbulent diffusivity fields and the abiotic DMS loss rates were used to compute net biological DMS production (NPBIO; see Materials and Experimental Methods) at high vertical-temporal resolution by budgeting (Fig. 6). In September, NPBIO rates (irrespective of the sign) were higher at the surface, decreasing with depth. Surface NPBIO varied approximately between −0.1 and 0.3 nmol L^−1^ h^−1^, with positive values (net production) during the mid-day, and negative values (net consumption) in the night. In May, NPBIO rates ranged from −0.03 to 0.7 nmol L^−1^ h^−1^ and were again at its highest at the surface (<5 m) during the day. Vertical gradients in NPBIO were less clear in May.

We compared model-computed NPBIO rates at the surface with those measured by incubating surface seawater on the deck (under mimicked upper mixed layer irradiance; see Materials and Experimental Methods) or incubated *in situ* (overboard) for 6–8 h during the September cruise. The results show remarkable agreement in the magnitude of daytime NPBIO (Supplementary Table S1) and the vertical NPBIO gradient (Supplementary Table S2).

## Discussion

The results of this study reveal the strong effects of mesoscale meteorological forcing on DMS production and concentration in the surface sea. They also illustrate why diurnal cycling and finely resolved vertical distributions, both resulting from water mixing dynamics and sunlight-synchronized microbial activities, must be taken into account when intending to predict ocean emissions of DMS.

### The importance of resolving vertical profiles

A notable difference between the two cruises was that in September the maximum DMS concentration was near the surface, whereas in May there was a subsurface maximum below the mixing layer depth. Thermocline-associated DMS maxima are quite common in the open ocean^18^,^31^,^33^,^38^,^39^,^40^. They may occur when gross DMS production occurs across the entire photic zone but, in the mixed layer, it is counteracted by strong losses by photolysis and ventilation^32^. As shown here and also in ref. 31, subsurface DMS peaks can supply new DMS into the mixing layer during mixing events, thereby contributing to emissions that would not have been predicted from surface measurements at coarser temporal resolution.

### Susceptibility to and resilience from meteorological perturbation

In September, the passage of a windstorm and subsequent return to the previous calm conditions provided the opportunity to monitor the effects of the perturbation and the resilience of the DMS-producing ecosystem. During the days prior to the storm, prolonged high insolation under weak wind caused gradual DMS accumulation. Then, the persistent wind reduced the surface DMS concentration to one third, mainly through ventilation to the atmosphere but also by turbulent mixing to deeper water. The overall loss of the DMS burden integrated down to 30 m depth (the deepest extent of wind-driven mixing according to temperature profiles) was in the order of 30%. Noteworthy, a similar coupling of DMS variability to synoptic-scale meteorological forcing had been observed in an early summer coccolithophore bloom in the North Atlantic^41^. In the present study, DMS concentrations recovered the pre-perturbation levels and diel pace in 3–4 days. Fluorescence profiles 2–3 days after the end of the storm showed enhanced phytoplankton growth, probably as a result of mixing-induced injection of either nutrients or cells from below. DMSPt concentrations at intermediate depths also increased towards the end of the cruise. However, surface DMS did not shift to a new regime in terms of concentration ranges and diel pace, but rather returned to the previous state. In order words, even though there was evidence for a weeklong shift of biomass and composition of the phytoplankton assemblage, in the short-term the DMS-producing system exhibited strong resilience.

### Wind forcing on DMS concentration and emission

The issue of mixed layer dynamics is relevant to understand DMS dynamics in the global oceans both at present and in response to global warming. One might think that mid and long-term changes in wind speed are first order drivers of trace gas emissions because gas transfer velocities are strongly dependent on wind speed. However, the magnitude of emission fluxes depends on trace gas concentrations as well, and persistent wind depletes concentration from the ocean by ventilation; besides, windy regions generally have deeply mixed waters where trace gases like DMS can hardly build up as they do in stratified waters^19^. Therefore, the net sign of wind speed shift effects on air-sea exchange of trace gases is not intuitive and depends on the timescale considered. Our results indicate that, under conditions favourable for DMS production (e.g., highly irradiated spring and summer waters), short-lived wind events may cause pulses of large emissions if there is enough time between events for the system to recover and DMS concentration to build up. The bottom line is that not only the changes in the average wind speed matter for forecasting DMS emission, but also the changes in the frequency of pulsed wind events.

### The DMS budget in the surface sea

**Figure 7:**
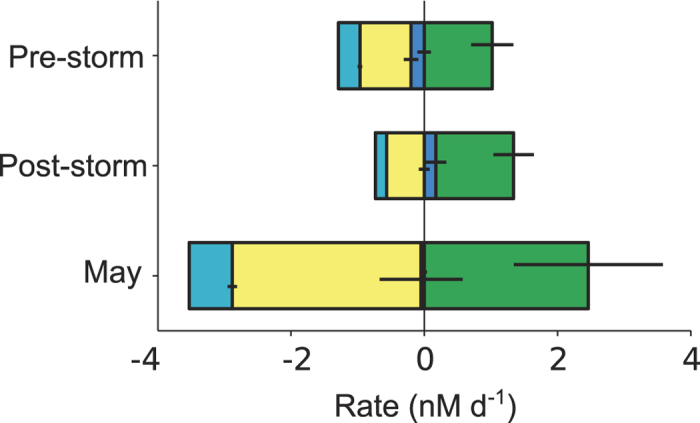
Average mixed-layer budgets of DMS source (positive) and loss (negative) process rates and their associated uncertainties in the three intensive studies. The 48-h averages are shown for ventilation (light blue), photolysis (yellow), vertical transport (dark blue) and net biological production (green). Numbers are given in Table S4.

With the detailed data obtained by observations and modelling in the intensive studies, we can attempt the 48-h average budgets of the process rates that control DMS concentrations in the UML (Fig. 7 and Supplementary Table S4). Biological processes were the largest DMS source in the three case studies, and also contributed the largest uncertainties to the budget. Vertical transport across the base of the UML was a minor source in September post-storm, and a minor UML loss in the pre-storm and May. Photolysis (PHOTO) largely surpassed ventilation (VENT) as a DMS sink. Assuming that gross DMS production (GP) refills the UML DMS pool once a day^17^ (that is, gross production in nmol L^−1^ d^−1^ equals DMS concentration in nmol L^−1^), then microbial DMS consumption (BC) can be estimated roughly as BC = GP - NPBIO and compared with the other loss rates. In September before the storm, BC accounted for some 37% of the daily DMS loss, PHOTO represented 38% of the loss and VENT and transport were 16% and 9%, respectively. After the storm, these figures were 36%, 49% and 15%, and average transport resulted in DMS injection into the UML. In May, loss shares were 50% (BC), 40% (PHOTO), 9% (VENT) and 1% (TRANSPORT). In these shallow MLs, therefore, microbial consumption and photolysis competed as the main loss processes, well above export into air above or water below. Photolysis rates equal or higher than microbial consumption rates had already been reported in the North Atlantic, also coinciding with shallow ML and high irradiance^41^. A recent meta-analysis compilation^17^ depicted similar figures for BC (ca. 40–50% of total loss), PHOTO (40–50%) and VENT (10–20%) in highly irradiated UML waters all over the globe.

### Sunlight forcing on DMS concentration and resulting diel cycles

Sunlight exerts a strong influence on the distribution and biogeochemical cycling of DMS^16^,^24^,^42^. Over the last decade, a number of works have suggested that the daily averaged solar radiation dose received in the upper mixed layer is a key factor governing DMS dynamics at all spatial scales, from the local to the global (see ref. 17 and references therein). Other works have reported a suite of experimental lines of evidence and hypothetical mechanisms whereby this emergent property occurs: the synergistic effects of UVR on microbial community DMS production, by enhancing phytoplankton DMSP and DMS release^17^,^42^,^43^,^44^,^45^, and simultaneously increasing the bacterial DMS production yield from dissolved DMSP consumption^46^,^47^ while inhibiting bacterial DMS consumption^16^. That is, UVR exposure stimulates DMS production and reduces its biological consumption, thereby driving increased net biological production. Increase of UVR-induced photochemical DMS destruction^48^ acts in the opposite direction, buffering DMS concentration in the short term^33^,^45^.

Plankton exposure to sunlight largely depends on meteorology, which governs irradiance and upper-ocean mixing. It also depends on the length and amplitude of the day-night cycles, set by latitude and season. Sunlight exposure modulates plankton activity not only by fuelling photosynthesis but also by causing photobiological stress and damage^49^,^50^. Phytoplankton coping with photobiological (oxidative) stress is one of the hypothetical explanations for the stimulation of DMS production by sunlight^43^. Our results show indeed that near surface, net biological production was positive and larger in the central hours of the day on both cruises. This coincided with indicators of photobiological stress in phytoplankton, such as decreased Φ_PSII_(Fig. 5; ref. 51) and increased ratios of diatoxanthin (a fast-responsive photoprotective pigment; ref. 52) to chl*a* (Supplementary Fig. S3).

Actually, in the first hours of the day, between dawn and noon, the concentration of DMSP decreased in the three intensive studies, coinciding with a decrease of Φ_PSII_ and increase of c_p_ (Fig. 5). After noon, DMSPt always increased, indicating net production. This is consistent with culture experiments conducted with the high DMSP producing haptophyte *Emiliania huxleyi*^44^. When a strain acclimated to daytime irradiances similar to those in the surface ocean was exposed to increased light, its intracellular DMSP content decreased in believed association with oxidative stress. When stress was relieved by dramatically reducing light intensity (the so-called ‘recovery phase’), the intracellular DMSP pool augmented again^44^. Therefore, DMSP decrease in the morning can be related to oxidative stress that converted DMSP into DMS^43^. Although this conversion by algal and bacterial DMSP lyases could resume in the afternoon, it was overcome by *de novo* DMSP biosynthesis under stress relief (Fig. 5), until phytoplankton mortality took off in the night.

Day-night oscillations in DMS concentration resulted from subtle short term imbalances among production and loss processes, as indicated by the opposite patterns encountered in September and May. Common to both cruises was the fact that biological DMS production overcame biological consumption near the surface during the hours of maximal insolation, resulting in positive NPBIO. In September, this was enough to cause daytime DMS accumulation in the upper mixed layer. Where sunlight-driven production remitted, namely below the mixed layer or throughout the water column at night, bacterial consumption^53^ took over to attenuate net DMS production or turn it into net loss. In May, however, sunlight-driven biological DMS production at the very surface was insufficient to overcome losses by photolysis, ventilation and bacterial consumption throughout the deeper mixed layer, and DMS decreased during the day. When photolysis shut down at night, DMS increased probably related to herbivore grazing and bacterial DMSP-to-DMS conversion. As shown in Supplementary Table S3, DMSPt consumption rates -which account for grazing-induced DMSPt loss^17^ - showed pronounced day vs. night differences in May but not in September. All in all, the corollary is that there is not such a thing as a universal diel pattern in DMS concentrations, as a recent circumnavigation study has concluded using a different approach^54^.

The most striking of our observations is how sharply the aforementioned imbalances change into reverse net effects around dawn and dusk. This points out to the involvement of circadian clocks in DMS cycling. Sunlight acts as the stimulus for on/off metabolic switches in plankton microbes, as recently revealed by metatranscriptomic analyses of open ocean samples^55^,^56^. Tight circadian control of physiological functions allows microbes to optimize cellular functions and to coordinate metabolic activities at the community level, which provides an advantage in the oligotrophic open ocean^57^. Coupling between autotrophs and heterotrophs is possibly driven by phytoplankton activity cycles^56^ and involves heterotrophic bacteria that use phytoplankton-released metabolites^57^, but also protist grazers^58^,^59^ and even viruses^58^. These coupling/decoupling cycles are thought to exert diel rhythmic forcing on biogeochemical fluxes^60^; however, observations like ours, showing diel rhythms in open-ocean biogeochemical processes, are very scarce^33^,^57^,^61^.

In summary, the high-resolution field results reported here align with the aforementioned previous observations of the strong influence of solar radiation onto the DMS cycle, and reveal for the first time that this influence operates in the short term, sub-daily to weekly. First, surface DMS concentration increased gradually in sunny calm days, even after a strong perturbation. Second, DMS concentrations peaked upper in the water column than those of DMSP and chl*a*. Third, DMS concentrations followed a 24 h period, with trend reversal points coinciding with switching points of the daily solar cycle and indicators of circadian rhythms. Fourth, positive net biological DMS production at sea surface occurred mainly during the day (noon and afternoon), coinciding with indicators of photoacclimation.

This study illustrates the value of detailed automated profiling in a Lagrangian sampling framework in terms of revealing the dynamics controlling DMS concentrations and fluxes and the intimate links between plankton photophysiological clocks and the temporal cycles of DMS. The results highlight the causes of spatial/temporal variability^54^,^62^ and its importance for understanding ecological processes and ocean/atmosphere fluxes of biogenic gases.

## Experimental Methods

### Study site and sampling scheme

The two cruises were conducted aboard R/V García del Cid in the Western Mediterranean Sea, in September 2011 and May 2012. Three Lagrangian drifters were deployed to track the movement of the upper 15-m water layer. Each drifter consisted of a spherical floatable enclosure that contained a GPS and an emitter, from which 10 m cylindrical drogues hanged 5 m below the sphere. We decided to track the upper 15 m water layer because, on the basis of previous cruises, we expected the mixing layer depth to be between 5 and 20 m. The drifters sent their position every 30 minutes, and all ship operations were conducted next to them. Both cruises were held in the same region, ca. 45 nautical miles from the coast, within the core of a cyclonic eddy over a water-column depth of ca. 2000 m (Supplementary Fig. S1). Seawater was withdrawn from 4 m depth using the ship’s underway pump, and measured continuously for DMS and phytoplankton fluorescence characteristics. Several times a day, a CTD probe equipped with a Niskin bottle rosette was used to profile the hydrographical properties until 200 m, and to collect discrete water samples along the vertical profile. On three occasions, (13–14 and 21–22 September, and 23–24 May), intensive studies were conducted over periods of approx. 30–40 hours. These consisted of CTD probing and sampling every 4 hours, and vertical underway profiling during the periods in between. The vertical profiler has been described in detail elsewhere^63^. Briefly, it consists of a small CTD probe with an attached tube connected to a peristaltic pump on deck. The CTD is operated in yoyo mode between surface and 35–50 m, at a descending-ascending speed of 2.5 to 4 m min^−1^.

### Meteorological measurements

Solar radiation and wind speed, among other variables, were measured with an Aanderaa Instruments automated meteorological station on board. A Solar Radiation Sensor 2770 provided global radiation in the wavelength range 0.3–2500 μm. Wind speed and wind direction were measured with a three-cup Sensor 2740/2740EX and a wind vane 3590/3590EX, respectively.

### Oceanographic measurements

A membrane equilibrator, atmospheric pressure chemical ionization mass spectrometer (Eq-APCIMS) was used for continuous DMS measurements from surface waters and across the vertical column during intensive studies (Supplementary Fig. S2). The instrument is described in detail in ref. 64 and the complete setup in ref. 63. Essentially, the Eq-APCIMS provided noise-filtered DMS concentrations every 30 s, which corresponded to depth steps of 1.3–2 m when profiling. The detection limit was nearly 0.1 nmol L^−1^, and precision was around 10%. Total (particulate and dissolved) DMSP concentration was measured in 3 mL discrete samples by gas chromatography (GC) coupled to flame photometric detection (FPD)^45^. The detection limit was nearly 3 pmol S and precision was around 5%. Chl*a* concentrations were determined by filtration of 150 mL of seawater through GF/F, extraction in acetone (90% v:v in water, 4 °C, overnight) and measurement in a Turner Designs fluorometer. Taxonomic and photoprotective pigments were analysed by HPLC (Spectra SYSTEM, Thermo) after GF/F filtration of 1–2 L of seawater and extraction with methanol^65^. A Fast Repetition Rate Fluorometer (FRRf, Chelsea Instruments) was used in continuous in the underway and the vertical profiler flows. Chl*a* fluorescence data (steady state *in vivo*, Fs, and maximum yield, Fm) were derived from the fluorescence induction curve^66^ and used to derive the relative efficiency of excitation energy captured by PSII, which was calculated as Φ_PSII_ =  (F′m − Fs)/F′m, where Fs is the steady state *in vivo* Chl*a* fluorescence of phytoplankton and F´m is the maximum yield of fluorescence during the illumination. This correlated with variations in the quantum yield of photosynthesis. Every day around noon, a PRR-800 multichannel profiling radiometer (Biospherical) was deployed overboard to profile underwater irradiances in the PAR, UVA and UVB bands. Extinction or diffuse attenuation coefficients of downward irradiance (Kd) were calculated as the linear regression between ln-transformed spectral irradiance and depth (z) in the optically homogeneous surface layer. All relevant optical data are described in detail elsewhere^67^. Seawater potential density (σ_t_) was calculated using high resolution CTD profile data. This was used to compute vertical profiles of the Brunt-Väisälä buoyancy frequency (s^−1^). High-resolution profiles were used to compute the mixing and mixed layer depths, defined by a given density difference (Δσ_t_) from the reference depth (2 m). A Δσ_t_ of 0.05 kg m^−3^ best matched the base of the high-turbulence mixing layer (Fig. S3); a Δσ_t_ of 0.125 kg m^−3^ was used to identify the less variable mixed layer depth, which matched the top of the pycnocline as depicted by the buoyancy frequency (Fig. 3).

### DMS cycling incubation experiments

To determine microbial DMS production and consumption rates, two UV-transparent Teflon bottles were filled with unfiltered seawater and incubated for 7–9 hours under the light in a black tank flushed with surface sea water to keep *in situ* temperature. One layer of a neutral mesh (60% transmittance) was used to simulate the average irradiance of the upper mixed layer. DMS concentration was measured at the beginning and end of the incubation. Changes in DMS concentration, once corrected for photochemical loss, corresponded to net biological production rate^45^. On two occasions, duplicate Teflon bottles were incubated under *in situ* light fields at different depths and NPBIO was calculated similarly (Supplementary Table S2). DMS photolysis rates were determined in surface seawater gravity filtered through 0.2 μm Nylon membrane. The filtrate was kept in the dark overnight to allow DMS concentrations to stabilize (i.e. until dissolved DMSP cleavage by lyase enzymes present in the filtrate stopped). UV transparent quartz bottles were filled with the filtrate and incubated in the outdoor tank with no neutral screen attenuation, and sacrificed in pairs after 4 h and 8 h of exposure. Two further replicates darkened with aluminium foil served as controls. A PUV-2500 multichannel radiometer (Biospherical) was placed at the centre of the incubation tank covered, as the samples, by 5 cm of water to keep a continuous record of spectral irradiance.

### Turbulent diffusivity modelling and DMS cycling equations

The General Ocean Turbulence Model (GOTM, www.gotm.net) was used to compute vertical profiles of turbulent diffusivity during the 3 intensive experiments (Supplementary Fig. S4). Realistic atmospheric forcing was applied using real observations of wind speed and direction, pressure, air temperature and humidity, and irradiance. Clouds were neglected for lack of data, and there was no precipitation. The 2nd order turbulence closure scheme was used with coefficients from ref. 68 and a k-ε style equation with a vertical resolution of 1 m and a time step of 10 s. The model was allowed to relax toward real *in situ* observations of temperature and salinity from the CTD of the continuous vertical profiler using a 30 min relaxation time. Observed DMS concentration profiles were gridded onto the model grid by interpolating DMS concentrations to 1 m (vertical) spatial resolution and 1 min temporal resolution. They were subsequently smoothed using the running average method.

At any time (*t*) and depth (*z*), DMS concentration is the net result of production and consumption processes:


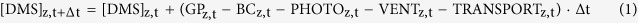
where GP = gross DMS production; BC = bacterial DMS consumption; PHOTO = DMS photolysis; VENT = DMS ventilation; TRANSPORT = DMS displacement by vertical turbulent diffusivity.

It should be noted that like all 1D turbulence models, the representation of internal waves and their contribution to vertical mixing is only approximated by GOTM using empirical values.

Vertical transport by non-turbulent advection is considered negligible. Since our model is 1D, we do not have any information on the 3D velocity structure of the eddy (including its vertical velocities). Some *in situ* measurements on similar anti-cyclonic eddies^69^ suggest that vertical non-turbulent velocities in an eddy are of the order of 3–4 m per day. This means that the Peclet number for such an eddy with our diffusivities (of up to 0.1 m^2^ s^−1^) would be of the order of 0.01. This in turn implies that vertical transport by diffusivity is 2 orders of magnitude larger than by non-turbulent advection. Based on this calculation it would seem justified that we chose to neglect non-turbulent transport.

A horizontal transport term is not included because the same water patch was sampled in Lagrangian mode over the entire duration of the cruises, and therefore horizontal advection was set to zero by definition. Besides, horizontal diffusivity was assumed negligible because strong horizontal gradients were not expected. We did not map the region for DMS concentration, so, we cannot prove this assumption to be true, but support is to be found in ref. 54: in these latitudes across the major oligotrophic ocean basins, there is little spatial variability in DMS concentration at scales <30 km. The entire straight distance covered in our cruises was ca. 20 km. All in all, our Lagrangian setting and the characteristics of our studied water masses allow us to assume that there was no significant horizontal transport of DMS, even if small deviations from purely Lagrangian sampling occurred due to, e.g., wind-driven ship drift between buoy re-visits.

Ventilation applies only to the very upper water layer, from where its effects are “redistributed” by turbulent diffusion. GP and BC can be merged into a single term called Net Biological DMS production (NPBIO):


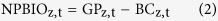


For the calculation of DMS photolysis (PHOTO), total surface irradiance (E_d,o,t_) and the underwater light extinction coefficients (K_d_) were combined to obtain the amount of radiation available at each *t* and *z* (E_d,z,t_):


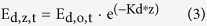



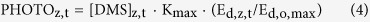
where K_max_ = photolysis rate constant at the water surface (details in ref. 70), and E_d,o,max_ = maximum irradiance at the water surface. The K_d_ at 330 nm (0.15 m^−1^) was used for downward photolysis propagation because the spectral peak of DMS photolysis in the UML has its maximum yield at this wavelength^39^. Emission or ventilation fluxes (VENT) were obtained as the product of DMS concentration in seawater and the transfer or piston velocity (k_w,DMS_, cm h^−1^):


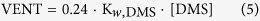


K_w__,__DMS_ was computed using wind speed data from the ship’s meteo station, SST and surface DMS concentrations, following ref. 71:


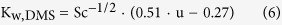
where u_10_ = wind speed at 10 m (m s^−1^) and Sc = Schmidt number of DMS, calculated from SST^72^. With high resolution data available for DMS concentration, wind speed and SST, emission fluxes were computed every minute, and then averaged every 30 minutes. DMS transport was calculated from vertical turbulent diffusivity (K_z_) diagnosed with the GOTM and vertical DMS gradient as:


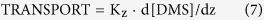


The final depth-time matrix of NPBIO was then obtained applying the following equation at a resolution of 1 m (vertical) and 1 min:


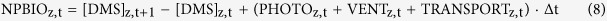


### Mean UML budgets and uncertainties

We assume that the main sources of uncertainty are DMS measurements themselves and photolysis, the loss term that drives the budgets. This justifies not taking into account uncertainties derived from sea-air flux and vertical mixing parameterizations. Uncertainty in 2-m and 30-min bin DMS concentration averages, as calculated by ref. 63, is CV_DMS = 11% (CV = coefficient of variation).

#### Ventilation

We assume no uncertainty in the sea-air flux parameterization. Note however that different parameterizations may differ by>50%.


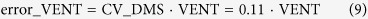


#### Photolysis

The CV of maximal subsurface photolysis rate constants (CV_Kmax) is 9% (SUMMER1) and 19% (SUMMER2). We can assume that uncertainties in irradiance cancel each other, since photolysis is vertically propagated following the quotient Ed,z,t/Ed,0,max. Thus, the uncertainty associated with the photolysis rate is calculated by adding the CVs in quadrature:


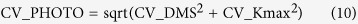



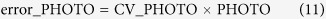


#### Transport

CV_DMS of 11% becomes 15.5% when computing the DMS gradient (dDMS = DMS_z1-DMS_z2), since errors add in quadrature.


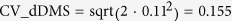


We assume that the error in Kz is the Kz range through a 2 m interval encompassing the ML base, which is the depth horizon used to compute DMS transport. CV_Kz is 48%, 88% and 86%, respectively, for pre-storm, post-storm and May intensive studies.


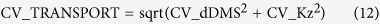



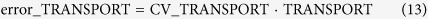


#### NPBIO

Error is propagated by adding in quadrature the error of loss terms plus the error in dDMS, the DMS change between two time steps, which is 15.5%.


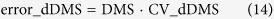



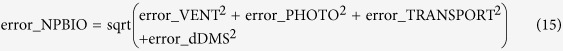


## Additional Information

**How to cite this article**: Royer, S.-J. *et al*. A high-resolution time-depth view of dimethylsulphide cycling in the surface sea. *Sci. Rep.* **6**, 32325; doi: 10.1038/srep32325 (2016).

## Supplementary Material

Supplementary Information
