# Supplementary material for: A high-resolution time-depth view of dimethylsulphide cycling in the surface sea
Source: Sci Rep. 2016 Aug 31;6:32325. doi: 10.1038/srep32325 (PMC5006029; doi:10.1038/srep32325)
Supplement: Supplementary Information [file srep32325-s1.pdf]

A high-resolution time-depth view of dimethylsulphide cycling in the surface sea

Supplementary Information

S-J. Royer[1,2], M. Galí[1,3], A.S. Mahajan[4], O.N. Ross[1,5], G.L. Pérez[6], E.S. Saltzman[7], R. Simó[1*]

1. *Institut de Ciències del Mar, CSIC, Passeig Marítim de la Barceloneta 37-49, 08003 Barcelona, Catalonia, Spain*
2. *Department of Oceanography, School of Ocean and Earth Science and Technology, University of Hawai'i at Mānoa, 1950 East-West Road, 96822, Honolulu, Hawaii, USA*
3. *Takuvik Joint International Laboratory and Québec-Océan, Université Laval, G1V OA6, Québec, QC, Canada*
4. *Indian Institute of Tropical Meteorology (IITM), Pashan Road, 411 008, Pune, India*
5. *Aix-Marseille University, CNRS, University of Toulon, IRD, MIO UM 110, 13288, Marseille, France*
6. *Instituto INIBIOMA (CRUB Comahue, CONICET), Quintral 1250, 8400 S.C. de Bariloche, Rio Negro, Argentina*
7. *University of California, Earth System Science Department, 3200 Croul Hall St, Irvine, California, 92697, United States*

Supplementary Table S1. Net biological DMS production rates (NPBIO) at surface waters in the two intensive studies during the September 2011 cruise, computed from diagnostic modelling, and determined experimentally from on-deck incubations. Data reported are a combination of before and after storm because similar rates and NPBIO diel cycles were found. Note that most incubations were conducted in the central daytime hours, matching the period of highest NPBIO. For diagnosed high-resolution NPBIO, the range refers to the 95% central values (gridded NPBIO at 30 minutes and 1 m intervals) within the upper mixed layer (with irradiance similar to that of the incubations). For the incubations, the range includes all measurements (if N > 1). N is the number of incubations (all run in duplicate 2.3 L UV-transparent Teflon bottles). Units are nmol DMS L^{-1} h^{-1}.

Cruise	Period of the day	Diagnosed NPBIO		NPBIO from incubations		
		Mean	Range (95%)	Mean ± sd	Range (all)	N
Sep 2011	7:30-16:30	0.15	0.04 - 0.30	0.093 ± 0.042	0.05 - 0.16	5
	15:30-22:30	-0.006	-0.07 - 0.14	0.011	0.011 - 0.012	2
	23:30-6:30	-0.03	-0.10 - 0.03	0.036		1

Supplementary Table S2. Vertical variation of net biological DMS production rates (NPBIO) from the intensive studies of September 2011, computed from diagnostic modelling, and determined experimentally from in-situ incubations at different depths. All incubations were run in duplicate 2.3 L Teflon bottles hanging overboard at different depths in the water column between 8 and 14 UTC. Units as in Table S1.

Experiment	Optically equivalent depth[a]	Diagnosed NPBIO		NPBIO from in situ incubations
		Mean	Range (95%)	Mean ± range
Pre-storm (16 Sep 2011)	2.5	0.16	0.04—0.26	0.10 ± 0.002
	6	0.12	0.03—0.19	0.09 ± 0.002
	13	0.05	-0.01—0.09	0.02 ± 0.002
Post-storm (20 Sep 2011)	2.5	0.18	0.10—0.24	0.16 ± 0.0005
	7	0.15	0.08—0.22	0.09 ± 0.0002
	20	0.07	0.03—0.11	0.04 ± 0.0008

[a]As defined in ref. 70

Supplementary Table S3. Total DMSP (DMSPt) consumption rates, calculated from the product of initial DMSPt concentration and the DMSPt consumption rate constant k_{DMSPt}. The latter was obtained from linear regression of ln[DMSPt] versus time in duplicate incubations of whole seawater samples in the dark. The linear regression R^2 in each incubation bottle was generally >0.95. Uncertainties include the range of k_{DMSPt} between duplicate bottles (generally ~7%) and the DMSPt analytical error (~5%). All incubations run in duplicate 2.3 L darkened Teflon bottles.

Cruise	Period	Rate (nmol L^{-1} h^{-1})
September 2011 (post-storm)	Day<br>Night	1.59 ± 0.29<br>1.53 ± 0.07
May 2012	Day<br>Night	0.23 ± 0.05<br>2.53 ± 0.16

Supplementary Table S4. Mean concentration, daily rates and their uncertainties in the upper mixed layer during each intensive diel cycle study. Rates in nmol L^{-1} d^{-1}. Note that e_ refers to estimated error in the corresponding rate (±).

**Variable**	September pre-storm	September post-storm	May
**Mean DMS (nmol L^{-1})**	1.78	1.59	5.97
**Ventilation**	0.32	0.17	0.65
**e_vent**	0.04	0.02	0.07
**rate_photolysis**	0.77	0.56	2.83
**e_photolysis**	0.11	0.08	0.62
**rate_transport**	0.20	-0.17	0.05
**e_transport**	0.10	0.16	0.04
**rate_NPBIO**	1.02	1.17	2.46
**e_NPBIO_sums**	0.32	0.30	1.12

Supplementary Figure legends

Supplementary Figure S1. September 2011 (pre- and post-storm) and May 2012 cruise trajectories overlaid on the bathymetric map of the Catalan Sea in the NW Mediterranean. The cruise trajectories were defined by the trajectories of the Lagrangian drifters. These maps have been created with the Ocean Data View software (Schlitzer, R., Ocean Data View, odv.awi.de, 2015).

Supplementary Figure S2. Vertical profiles of the ratio of diatoxanthin to chlorophyll *a* in biomass samples from September (dark green diamonds) and May (light green triangles). Open symbols correspond to midday measurements (10:00-15:00) and filled symbols correspond to night measurements (dusk to dawn).

Supplementary Figure S3. Vertical profiles of turbulent diffusivity as computed with the GOTM model for the three intensive studies.

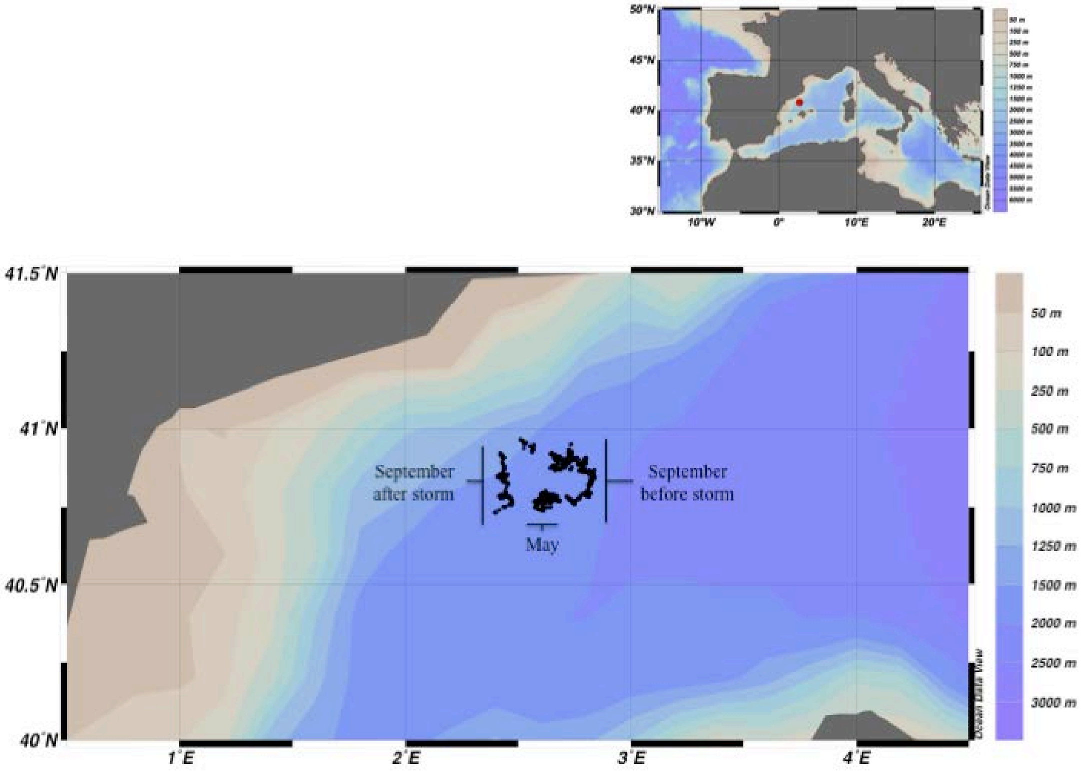

Supplementary Figure S1. September 2011 (pre- and post-storm) and May 2012 cruise trajectories overlaid on the bathymetric map of the Catalan Sea in the NW Mediterranean. The cruise trajectories were defined by the trajectories of the Lagrangian drifters. These maps have been created with the Ocean Data View software (Schlitzer, R., Ocean Data View, odv.awi.de, 2015).

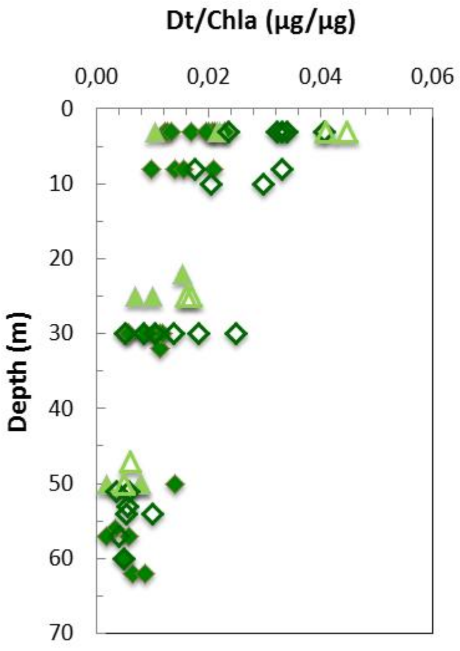

Supplementary Figure S2. Vertical profiles of the ratio of diatoxanthin to chlorophyll *a* in biomass samples from September (dark green diamonds) and May (light green triangles). Open symbols correspond to midday measurements (10:00-15:00) and filled symbols correspond to night measurements (dusk to dawn).

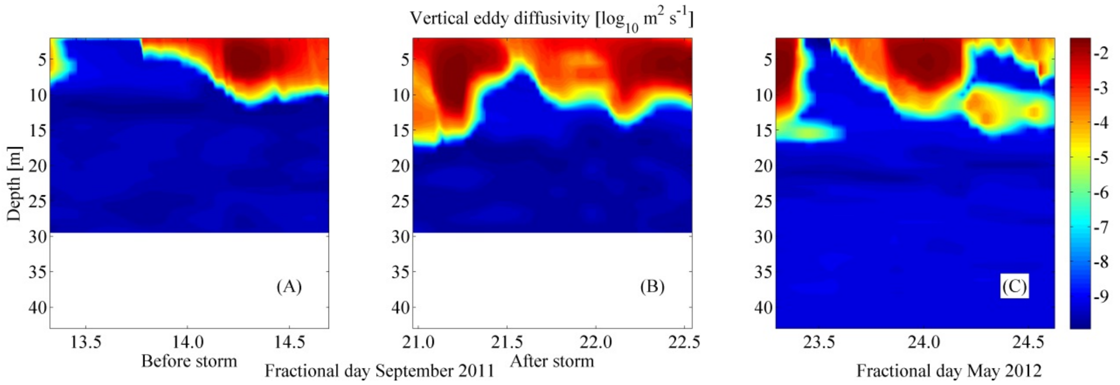

Supplementary Figure S3. Vertical profiles of turbulent diffusivity as computed with the GOTM model for the three intensive studies.
